# Increased Expression Levels of Netrin-1 in Visceral Adipose Tissue during Obesity Favour Colon Cancer Cell Migration

**DOI:** 10.3390/cancers15041038

**Published:** 2023-02-07

**Authors:** Amaia Mentxaka, Javier Gómez-Ambrosi, Gabriela Neira, Beatriz Ramírez, Sara Becerril, Amaia Rodríguez, Víctor Valentí, Rafael Moncada, Jorge Baixauli, María A. Burrell, Camilo Silva, Vasco Claro, Albert Ferro, Victoria Catalán, Gema Frühbeck

**Affiliations:** 1 Metabolic Research Laboratory, Clínica Universidad de Navarra, 31008 Pamplona, Spain; 2 CIBER Fisiopatología de la Obesidad y Nutrición (CIBEROBN), Instituto de Salud Carlos III, 31008 Pamplona, Spain; 3 Obesity and Adipobiology Group, Instituto de Investigación Sanitaria de Navarra (IdiSNA), 31008 Pamplona, Spain; 4 Department of Surgery, Clínica Universidad de Navarra, 31008 Pamplona, Spain; 5 Department of Anesthesia, Clínica Universidad de Navarra, 31008 Pamplona, Spain; 6 Department of Histology and Pathology, Universidad de Navarra, 31008 Pamplona, Spain; 7 Department of Endocrinology & Nutrition, Clínica Universidad de Navarra, 31008 Pamplona, Spain; 8 School of Cardiovascular & Metabolic Medicine and Sciences, British Heart Foundation Centre of Research Excellence, King’s College London, London SE1 9NH, UK

**Keywords:** NTN-1, NEO-1, DCC, UNC5B, obesity, adipose tissue, inflammation, colon cancer, cell migration

## Abstract

**Simple Summary:**

Netrin-1 (NTN-1) regulates obesity-associated low-grade inflammation, being also involved in the control of cell migration and proliferation. We aim to study whether excess visceral adipose tissue in patients with obesity and colon cancer is associated with increased *NTN1* and the expression levels of its main receptors, promoting an inflammatory microenvironment that favours colon cancer development. Increased expression levels of *NTN1* and its receptor *NEO1* in the visceral adipose tissue from patients with obesity and colon cancer together with elevated *DCC* and *UNC5B* mRNA levels in patients with colon cancer were found. Moreover, the treatment of colorectal cancer cells with NTN-1 and with the adipocyte-derived secretome obtained from patients with obesity increased the migration of colorectal cancer cells. These results suggest that NTN-1 plays an important role in obesity-associated colon cancer development.

**Abstract:**

Netrin (NTN)-1, an extracellular matrix protein with a crucial role in inflammation, is dysregulated during obesity (OB) and influences colon cancer (CC) progression. To decipher the mechanisms underlying CC development during obesity, we examined the expression of *NTN1* and its receptors in the visceral adipose tissue (VAT) of 74 (25 normal weight (NW)) (16 with CC) and 49 patients with OB (12 with CC). We also evaluated the effect of caloric restriction (CR) on the gene expression levels of *Ntn1* and its receptors in the colon from a rat model fed a normal diet. The impact of adipocyte-conditioned media (ACM) from patients with OB and NTN-1 was assessed on the expression levels of neogenin 1(*NEO1*), deleted in colorectal carcinomas (*DCC*) and uncoordinated-5 homolog B (*UNC5B*) in Caco-2 and HT-29 human colorectal cell lines, as well as on Caco-2 cell migration. Increased *NTN1* and *NEO1* mRNA levels in VAT were due to OB (*p* < 0.05) and CC (*p* < 0.001). In addition, an upregulation in the expression levels of *DCC* and *UNC5B* in patients with CC (*p* < 0.01 and *p* < 0.05, respectively) was observed. Decreased (*p* < 0.01) *Ntn1* levels in the colon from rats submitted to CR were found. In vitro experiments showed that ACM increased *DCC* (*p* < 0.05) and *NEO1* (*p* < 0.01) mRNA levels in HT-29 and Caco-2 cell lines, respectively, while *UNC5B* decreased (*p* < 0.01) in HT-29. The treatment with NTN-1 increased (*p* < 0.05) *NEO1* mRNA levels in HT-29 cells and *DCC* (*p* < 0.05) in both cell lines. Finally, we revealed a potent migratory effect of ACM and NTN-1 on Caco-2 cells. Collectively, these findings point to increased NTN-1 during OB and CC fuelling cancer progression and exerting a strong migratory effect on colon cancer cells.

## 1. Introduction

Colorectal cancer (CRC) constitutes the third most common diagnosed cancer and the second in mortality rates, with more than half of all cases and deaths being attributed to potentially preventable risk factors [1,2]. Particularly, overweight and obesity account for 11% of CRC cases in Europe, and excess body fat is associated with a relative risk of 1.3 of CRC development [1,2,3,4,5]. Different factors are considered to associate obesity and colon carcinogenesis, including increased insulin-like growth factor (IGF)-1 axis signalling, sex hormone alterations, gut dysbiosis, increased hypoxia and fibrosis, changes in the adipocyte-derived cytokines, and the low-grade chronic inflammatory state [6,7]. Of interest, netrin-1 (NTN-1), a multifunctional secreted glycoprotein member of the neuronal guidance proteins, mediates crucial functions in inflammation [8,9], as well as in cell migration and proliferation [10,11], by signalling through different receptors including neogenin-1 (NEO-1), deleted in colorectal carcinomas (DCC), and uncoordinated-5 homolog family members (UNC5A, UNC5B, UNC5C, and UNC5D) [12].

The involvement of NTN-1 in the regulation of obesity-associated pathologies has been extensively demonstrated [8,9,13,14]. NTN-1 is highly expressed in the adipose tissue (AT) of humans and animal models with obesity, regulating macrophage accumulation and promoting chronic inflammation and insulin resistance [14,15]. In addition, NTN-1 has been described as a survival factor in a wide range of aggressive cancers including metastatic breast, pancreatic, lung, or liver cancer [11,16,17]. NTN-1 also plays a critical role in CRC development [10,18]. Increased circulating levels of NTN-1 in patients with CRC have been reported [19], being highest in the last stages and during the invasive tumoral phases [11,20]. NTN-1 has been identified as a direct target of the transcription factor nuclear factor κ B (NFκB), strengthening its involvement as a mediator between inflammation and tumour development [21,22]. Furthermore, the upregulation of *NTN1* gene expression levels together with the loss of its tumour suppressor receptors *DCC* and *UNC5B* has been associated with the inhibition of cell death, leading to the spontaneous formation of gastrointestinal neoplastic lesions [10,20,22,23]. In this line, the expression levels of *Ntn1* were upregulated in the colon from mice containing a germline mutation in the adenomatous polyposis coli (*Apc)* gene, as well as in azoxymethane and dextran sulphate models of colitis-associated colon cancer (CC), inhibiting colonic epithelial cell apoptosis and promoting the development of high-grade colon adenomas [10,18].

Despite growing epidemiological studies supporting a causal association between obesity and cancer development, the molecular basis remains unclear. We hypothesised that dysfunctional visceral AT (VAT) in patients with obesity is associated with increased *NTN1* expression levels promoting an inflammatory microenvironment that favours CC development. Thus, we first investigated whether the expression of *NTN1* and its receptors are influenced by obesity and CC in human VAT. We further evaluated the effect of caloric restriction on *Ntn1* gene expression levels in the colon of a rat model. Subsequently, we compared the impact of the adipocyte-derived secretome from patients with obesity and NTN-1 on the expression levels of *NEO1*, *DCC*, and *UNC5B* in two human colorectal cell lines, Caco-2 and HT-29. Finally, the migratory effect of the adipocyte-conditioned media (ACM) and NTN-1 on Caco-2 cells was assessed in real time.

## 2. Materials and Methods

### 2.1. Study Population

Seventy-four (25 normal weight (NW) (16 with CC) and 49 patients living with obesity (OB) (12 with CC)) recruited from healthy volunteers and patients at the Clínica Universidad de Navarra were included in the study. The inclusion criteria were 18–65-year-old males and females, body mass index (BMI) between 18.5–24.9 kg/m^2^ for NW subjects and BMI ≥30.0 kg/m^2^ for volunteers with OB, absence of psychiatric pathology, validated diagnosis of CC for patients included in the CC group, and written informed consent for participation in the study. The exclusion criteria were infectious/inflammatory diseases, severe nephropathy, pregnancy or lactation, and people whose freedom is under legal or administrative requirement. Body weight was measured with a digital scale to the nearest 0.1 kg and height was calculated with a Harpenden stadiometer to the nearest 0.1 cm (Holtain Ltd., Crymych, UK) as previously described [24]. BMI was calculated dividing kilograms of weight by the square of the height in meters. The CUN-BAE formula was used to estimate body fat (BF) [25]. The pathological characteristics of the subjects with CC included in the study are displayed in Table S1.

The experimental design was approved, from an ethical and scientific perspective, by the Universidad de Navarra’s Ethical Committee responsible for research (2019.089). Informed consents were obtained voluntarily from all participants. The study was carried out in accordance with the Declaration of Helsinki’s ethical guidelines.

### 2.2. Surgical Procedures, Sample Collection, and Analytical Measurements

VAT samples were collected as previously reported [26] from patients undergoing Nissen fundoplication (NW volunteers, n = 9), Roux-en-Y gastric bypass (patients with OB, n = 37), and curative resection for primary colon carcinoma (patients with CC, n = 28).

Plasma and serum samples were obtained in the morning following a 12 h fast to ensure the absence of biochemical and hormonal alterations. Serum glucose, triglyceride, and C-reactive protein (CRP) concentrations were analysed by enzymatic spectrophotometric reactions using an automated analyser (Hitachi Modular P800, Roche, Basel, Switzerland). Circulating levels of the intestinal damage biomarkers calprotectin subunit S100 calcium-binding protein A8 (S100A8) (R&D Systems, Abingdon, UK), lactoferrin (Invitrogen, Carlsbad, CA, USA), and C-C motif chemokine ligand 5 (CCL5) (R&D Systems) concentrations were determined by commercially available ELISA kits following the manufacturer’s instructions. The mean intra-assay and inter-assay coefficient of variations were 3.4% and 4.6% for S100A8, 10% and 12% for lactoferrin, and 2.3% and 6.5% for CCL5, respectively.

### 2.3. Experimental Animals

Ten-week-old male Wistar rats (n = 40) (bred in the University of Navarra animal facility) were individually housed in a ventilated room (at least 15 complete air changes/h) with relative humidity (50 ± 10%), at a controlled temperature (22 ± 2 °C) and a 12:12 h light–dark cycle (lights on at 8:00 am). Rats were fed ad libitum with a normal chow diet (ND, n = 20) (Diet 2014S, Harlan Laboratories Inc., Barcelona, Spain) or a high-fat diet (HFD, n = 20) (Diet F3282, Bio-Serv, Frenchtown, NJ, USA) for 16 weeks as previously described [27]. The animals’ weight and food intake were registered on a weekly basis. Rats under ND and HFD were randomly assigned into two groups (n = 10 each) to analyse the effect of a 25% caloric restriction (CR) (ND/CR and HFD/CR). Following all procedures, rats were sacrificed by decapitation after an 8 h fast, and the colon was removed and stored at −80 °C until the next experiments. All experimental protocols adhered to the European Guidelines for the Care and Use of Laboratory Animals (directive 2010/63/EU) and were approved by the Ethics Committee for Animal Experimentation of the University of Navarra (049/10).

### 2.4. Human Adipocyte and Colon Adenocarcinoma Cell Cultures

Stromal vascular fraction cells (SVFC) from patients with OB were isolated from VAT and differentiated to adipocytes, as previously described [28]. To prepare the ACM, the supernatant of differentiated adipocytes was collected, centrifuged at 200 *g* for 15 min and frozen at −80 °C. Two colorectal adenocarcinoma cell lines, HT-29 (HTB-38^TM^) and Caco-2 (HTB-37^TM^), were provided by the ATCC^®^ (Washington DC, NW, USA) and cultured according to the manufacturer’s instructions. The Caco-2 cell line was kindly gifted by Dr. Amaya Azqueta from the University of Navarra. Briefly, HT-29 and Caco-2 cells were seeded at 3 × 10^5^ cells/well and grown in McCoy’s 5A medium with L-glutamine (Merck, Darmstadt, Germany) and DMEM/F-12 [1:1] (Invitrogen), respectively, supplemented with 10% of new-born-calf serum (Merck) and antibiotic–antimycotic (Invitrogen) at 37 °C for 72 h. Both types of tumour colorectal cells were serum starved for 2 h and then treated with ACM (20% and 40%) and NTN-1 (200 ng/mL and 500 ng/mL) (R&D Systems) for 24 h.

### 2.5. Analysis of Gene Expression Levels

Human VAT and rat colon samples were homogenised with an Ultra-Turrax^®^ T25 basic (IKA-Werke GmbH, Staugen, Germany). Total RNA was isolated using the QIAzol^®^ reagent (Qiagen, Hilden, Germany) for VAT and adipocytes, and the TRIzol^®^ reagent (Invitrogen) for colon samples, as well as Caco-2 and HT-29 cell lines. Samples were purified using the RNeasy Mini kit (Qiagen) and treated with DNase I (Qiagen) to remove genomic DNA, as previously reported [29]. For the cDNA synthesis, 2 µg of total RNA were transformed to cDNA using random hexamers (Roche) as primers and 200 units of M-MLV reverse transcriptase (Invitrogen).

Transcription levels of apoptosis-associated speck-like protein containing a CARD (*ASC*), *DCC*, *NEO1*, interleukin (IL)-1β (*IL1B*), *IL18*, NLR family pyrin domain containing 6 (*NLRP6*), *NTN1*, and *UNC5B* were quantified by Real-Time PCR (7300 Real Time PCR System, Applied Biosystem, Foster City, CA, USA). Primers and probes (Merck) were designed using Primer Express 2.0 software (Applied Biosystems, Foster City, CA, USA) (Table S2). The probes covered the ends of two exons to prevent amplification of the genomic DNA. The cDNA was amplified as previously described [30]. Primer and probe concentrations for gene amplification were 300 nM and 200 nM, respectively. The results were normalised to the levels of the ribosomal *18S* rRNA (Applied Biosystems) and relative quantification was calculated with the ∆∆Ct formula. Relative mRNA expression was expressed as a fold increase over the calibrator sample (average of gene expression corresponding to the NW group without CC, ND fed rats, or unstimulated cells), as previously described [31]. The mean values of the samples were calculated after they were analysed in duplicate.

### 2.6. Migration Assay

Caco-2 cell migration in response to NTN-1, ACM and IL-8 stimuli was measured with the electronic-impedance-based xCELLigence real-time cell migration assay system (Roche Applied Science, San Diego, CA, USA) monitoring every 5 min. Caco-2 cells were grown in DMEM/F-12 media (Invitrogen) supplemented with 2% FBS (Merck) at 37 °C for 24 h. Next, 1 × 10^5^ cells were directly placed on top of the 8 µm microporous polyethylene terephthalate (PET) membrane of the 16-well cell invasion and migration (CIM) plates (Roche Applied Science) previously covered with DMEM/F-12 media containing 2% FBS. Cells were serum starved for 2 h before treatment. The lower chamber was filled with DMEM/F-12 media containing 2% FBS with either NTN-1 (200 ng/mL and 500 ng/mL, R&D Systems), ACM (20% and 40%), or IL-8 (100 ng/mL, R&D Systems) as chemo-attractants for cells. The chemokine IL-8 was used as a positive control because of its known role in the migration of in vitro colon cancer cell models [32,33]. The impedance sensors automatically detected cells as they migrated and attached to the impedance microelectrodes in the lower chamber. The impedance of electron flow caused by adherent cells was reported using a unitless parameter called cell index (CI). After cell migration for 20 h, RTCA Software Pro version 2.2 (San Diego, CA, USA) was used to determine the number of cells that reached the lower chamber, as well as data display and analysis [34,35]. The experiment was repeated three times in quadruplicate.

### 2.7. Data and Statistical Analysis

Data are displayed as mean ± SEM. Due to the non-normal distribution, gene expression levels were logarithmically transformed. To examine the influence of our two categorical factors, namely OB and CC, two-way ANCOVA (age as covariable) was used. In the case of interaction between factors (*P* OBxCC < 0.05), one-way ANOVA followed by Tukey’s post hoc tests were applied. One-way ANOVA followed by Dunnett’s post hoc tests were used to evaluate differences in the in vitro experiments, and two-tailed unpaired Student’s *t*-tests were applied to analyse differences in the animal model. Pearson’s correlation coefficient (r) was used to analyse the association between variables. The calculations were performed with the SPSS/Windows version 15.0 statistical package (SPSS, Chicago, IL, USA), and graphs were generated with GraphPad Prism version 8.3 (GraphPad Software, Inc., San Diego, CA, USA). A *p* value <0.05 was considered statistically significant.

## 3. Results

### 3.1. NTN1 and NEO1 Expression Levels Are Increased in VAT in Obesity and CC and Are Associated with Key Inflammatory Factors

Table 1 shows the clinical features of the study population. Differences between groups were adjusted by age because of significant differences between CC and non-CC patients (*p* < 0.001). Weight, BMI, and estimated BF were increased (*p* < 0.01) in patients with OB compared to the NW volunteers. Although patients with CC exhibited higher glucose concentrations compared to patients without CC, differences did not reach statistical differences. Obesity was clearly associated with higher circulating levels of triglycerides (*p* < 0.01) and with the intestinal damage biomarkers S100A8 (*p* < 0.001) and lactoferrin (*p* < 0.05). Patients with OB without CC exhibited higher concentrations (*p* < 0.01) of CCL5 compared to the rest of the groups. Overall, the differences in leukocytes did not reach statistical significance.

**Figure 1:**
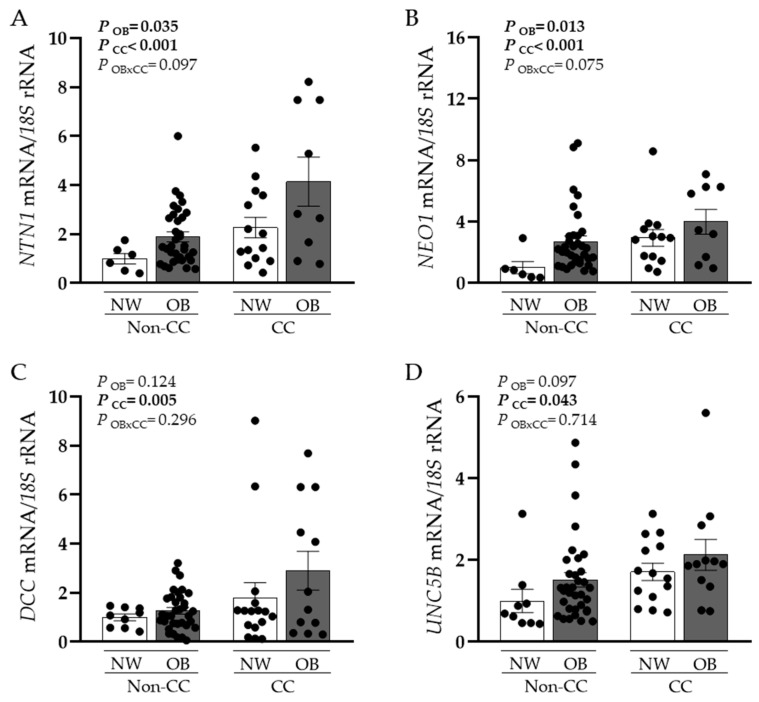
Gene expression levels of (**A**) netrin-1 (*NTN1*), (**B**) neogenin-1 (*NEO1*), (**C**) deleted in colorectal carcinomas (*DCC*), and (**D**) uncoordinated-5 homolog family member B (*UNC5B*) in visceral adipose tissue (VAT) from normal weight volunteers (NW) without (n = 6–9) and with (n = 14–16) colon cancer (CC) and patients with obesity (OB) without CC (n = 34–37) and with CC (n = 9–12). Bars represent the mean ± SEM. Differences between groups were analysed by two-way ANCOVA.

A significant upregulation in the gene expression levels of *NTN1* and *NEO1* in VAT caused by OB (*p* < 0.05) and CC (*p* < 0.001) was observed (Figure 1A,B). Although no effect of OB was found in the mRNA expression levels of *DCC* and *UNC5B*, a significant increase was found in patients with CC (*p* < 0.01 and *p* < 0.05, respectively) (Figure 1C,D). Since volunteers with OB without CC exhibited higher BMI and BF than patients with OB and CC, we also analysed differences between groups controlled by BF finding the same tendency. Furthermore, gene expression levels of *NTN1* and its receptors were positively associated between them (Table 2). Importantly, *NTN1* expression was also associated with glucose (r = 0.27, *p* = 0.046), as well as with the markers of inflammation CRP (r = 0.41, *p* = 0.020) and the percentage of monocytes (r = 0.37, *p* = 0.011). No association between *NTN1* mRNA levels and BMI was detected in our study, which may be due to the increased inflammatory profile of both groups of patients with CC, NW, and OB.

Given the importance of NTN-1 in inflammation, we also aimed to analyse its association with relevant genes involved in VAT inflammation, but also with a crucial function in intestinal homeostasis. mRNA levels of the inflammasome *NLRP6*, its associated molecule *ASC*, as well as its main effector *IL1B* were significantly increased because of OB and CC (Table 3). Interestingly, mRNA levels of *NTN1*, *NEO1*, and *UNC5B* were associated with *ASC* and *IL18* (Table 2).

### 3.2. Decreased Ntn1 Expression Levels in the Colon of Rats after Caloric Restriction

**Figure 2:**
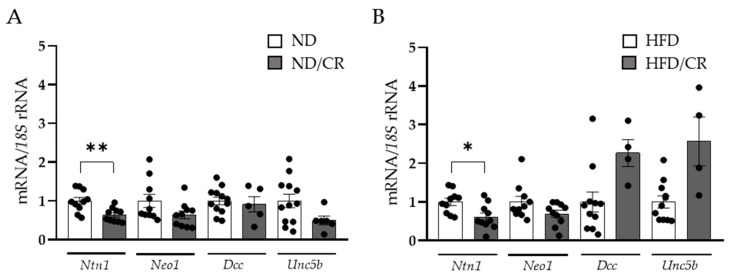
Effect of caloric restriction on netrin-1 (*Ntn1*), neogenin-1 (*Neo1*), deleted in colorectal carcinomas (*Dcc*), and uncoordinated-5 homolog family member B (*Unc5b*) gene expression levels in the colons of rats under a normal (ND) (**A**) or a high-fat diet (HFD) (**B**) (n = 4–12). Gene expression levels in rats fed an ND or an HFD were assumed to be 1. Values are the mean ± SEM. Differences between groups were analysed by two-tailed unpaired Student’s *t*-tests. * *p* < 0.05, ** *p* < 0.01.

To better understand the effect of weight loss on NTN-1 regulation in the colon, we analysed its expression levels and its receptors in a rat model under both ND and HFD, submitted to CR (Figure 2). Numerous studies support the beneficial effects of food restriction, alleviating different obesity-associated complications, including insulin resistance and systemic inflammation [36]. In our study, rats on a 25% CR exhibited decreased colonic *Ntn1* mRNA levels (*p* < 0.01) in rats fed an ND or an HFD together with a tendency to downregulate *Neo1, Dcc* and *Unc5b* expression levels remained unchanged.

### 3.3. Adipocyte-Conditioned Media and NTN-1 Induce the Expression of NEO1 and DCC in Human Colorectal Cells

**Figure 3:**
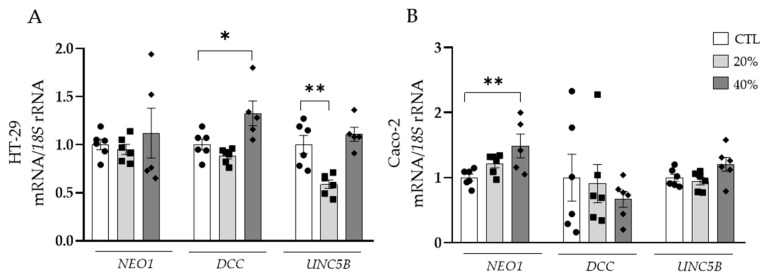
Gene expression levels of neogenin-1 (*NEO1*), deleted in colorectal carcinomas (*DCC*) and uncoordinated-5 homolog family member B (*UNC5B*) in (**A**) HT-29 and (**B**) Caco-2 human colorectal cancer cell lines after the treatment with adipocyte conditioned media (ACM) for 24 h. Gene expression levels in untreated cells were assumed to be 1. Values are the mean ± SEM (n = 4–6 per group). Differences between groups were analysed by one-way ANOVA followed by Dunnett’s post hoc tests. * *p* < 0.05 and ** *p* < 0.01.

Due to the potential contribution of dysregulated adipokines in tumour development, the effect of the secretome of adipocytes obtained from patients with OB on the expression levels of the main receptors of NTN-1 was investigated in both HT-29 and Caco-2 cell lines. After the treatment of HT-29 cells with ACM, an upregulation (*p* < 0.05) of *DCC* expression levels was observed (Figure 3A). Oppositely, *UNC5B* mRNA levels exhibited a significant decrease (*p* < 0.01), while *NEO1* expression levels remained constant (Figure 3A). *NEO1* expression levels were significantly upregulated (*p* < 0.01) in Caco-2 cells treated with ACM, whereas *DCC* and *UNC5B* remained unchanged (Figure 3B).

**Figure 4:**
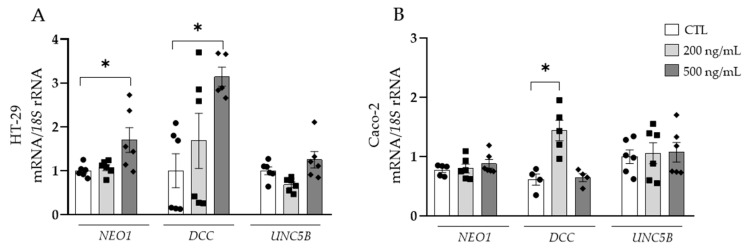
Gene expression levels of neogenin-1 (*NEO1*) deleted in colorectal carcinomas (*DCC*) and uncoordinated-5 homolog family member B (*UNC5B*) in (**A**) HT-29 and (**B**) Caco-2 human colorectal cancer cell lines after the treatment with netrin-1 (NTN-1) for 24 h. Gene expression levels in untreated cells were assumed to be 1. Values are the mean ± SEM (n = 4–6 per group). Differences between groups were analysed by one-way ANOVA followed by Dunnett’s post hoc tests. * *p* < 0.05.

Next, we studied the specific role of NTN-1, finding an upregulation of *DCC* in HT-29 and Caco-2 cell lines (Figure 4A,B). The mRNA expression of *NEO1* was increased (*p* < 0.05) in HT-29 cells and gene expression levels of *UNC5B* remained unchanged in both colorectal cancer cell lines.

### 3.4. Effect of Adipocyte-Conditioned Media and NTN-1 on Caco-2 Cell Migration

**Figure 5:**
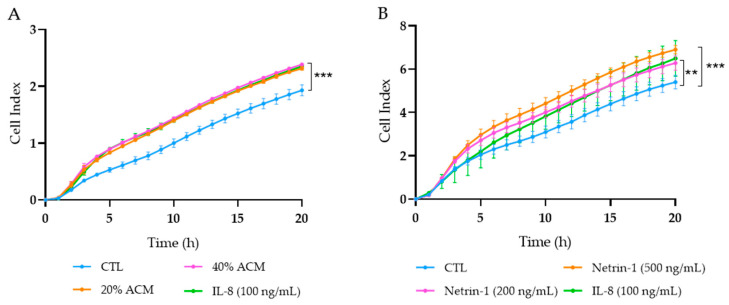
Migration of Caco-2 cells treated with (**A**) adipocyte conditioned media (ACM), (**B**) netrin-1 (NTN-1), and interleukin (IL)-8. RPMI media with 2% FBS was used as the baseline control (CTL), and IL-8 was the positive control of migration. Graphs represent the mean from four replicates in three different experiments. Differences between groups were analysed by one-way ANOVA followed by Dunnett’s post hoc tests. ** *p* < 0.01 and *** *p* < 0.001 vs. CTL.

Since dysregulated secretion of adipokines promotes cancer cell progression in adjacent tissues to adipose depots [37,38] and NTN-1 has been reported to trigger cancer cell migration [39,40], we aimed to elucidate the role of ACM and NTN-1 in the modulation of tumour Caco-2 cell chemotaxis. Caco-2 cells treated with ACM at 20% and 40% showed a significant increase (*p* < 0.001) in chemotaxis, similar to the migration observed after the stimulation with the positive control IL-8 (Figure 5A). After the treatment with NTN-1 at 200 ng/mL, Caco-2 cells reached a similar cell index migration rate as after the treatment with IL-8, both being significantly increased (*p* < 0.01) compared with the control cells (Figure 5B). After the stimulation with NTN-1 at 500 ng/mL, tumour cells exhibited a higher (*p* < 0.001) migration effect compared to untreated cells (Figure 5B).

## 4. Discussion

Independent studies have identified NTN-1 as a key tumorigenic factor in CC and as a pro-inflammatory player in OB, suggesting that its increase during OB may induce CC development [14,41]. To elucidate the mechanisms contributing to CC progression during obesity, we studied the expression of *NTN1* and its receptors in VAT and the effect of ACM and NTN-1 on colorectal cancer cell migration. We found increased *NTN1* and *NEO1* expression levels in VAT caused by OB and CC, whereas *DCC* and *UNC5B* mRNA levels were upregulated in patients with CC. We also found a downregulation of *Ntn1* levels in the colon from rats submitted to CR. Furthermore, both NTN-1 and ACM were found to increase *NEO1* and *DCC* mRNA levels in two colorectal adenocarcinoma cell lines, together with *UNC5B* downregulation in HT-29 cells when stimulated with ACM. Finally, Caco-2 cells exhibited a strong migratory effect after the treatment with ACM and NTN-1.

During obesity, the inflamed VAT is a source of pro-inflammatory adipokines including tumour necrosis factor (TNF)-α, IL-6, IL-8, CRP, or leptin, among others, that are released into the circulation promoting systemic inflammation [42,43,44]. Relevant studies have demonstrated obesity-associated inflammation as a crucial step in the establishment of the tumour microenvironment, conferring optimal survival conditions to dysregulated pro-tumorigenic cells [45]. Our group previously described that circulating concentrations and VAT gene expression levels of the pro-inflammatory mediator NTN-1 are increased in patients with OB being associated with insulin resistance [14]. For the first time, our findings show increased *NTN1* expression levels in VAT from patients with OB and CC. We also observed higher *DCC* and *UNC5B* expression in VAT, especially in patients with CC, as well as *NEO1* upregulation in OB and CC, suggesting that during tumorigenesis, the downstream signalling of NTN-1 is continuously activated, potentiating inflammation and cell survival. We further demonstrate a positive association between *NTN1* and its receptors, as well as with relevant proinflammatory genes involved in the maintenance of intestinal homeostasis including *NLRP6*, *ASC*, and *IL18*, endorsing the pro-inflammatory nature of NTN-1, similar to that of inflammasome products secreted mainly by activated macrophages. In this line, our group previously demonstrated that the treatment of visceral adipocytes with NTN-1 promoted the upregulation of proinflammatory and chemotactic molecules (*CCL2*, *IL1B*, *IL8*, and *IL36*) strengthening the role of NTN-1 in favouring VAT inflammation [14]. Patients with CC and inflammatory bowel disease, a condition involving a chronic inflammation similar to the VAT from patients with OB, showed upregulated levels of *NTN1* in the colon being related to tumour progression, suggesting that OB-associated VAT inflammation increased *NTN1* expression levels in CC favouring and supporting tumour development [18]. High *NTN1* expression levels in VAT during OB and CC may perpetuate the systemic inflammatory state favouring a microenvironment that impacts the colon and fuels CC development.

In light of previous studies showing increased *Ntn1* in the AT from obese mice, we aimed to determine whether CR influences *Ntn1* expression levels in the colon of ND-fed rats [15]. We found that *Nnt1* mRNA levels were downregulated in animals submitted to CR, strengthening the antitumoral effect of CR by reducing inflammatory signalling pathways [46,47]. In this regard, a study in high-fat- and low-fat-fed rats, as well as in azoxymethane-treated and control rats, found that CR decreased the proliferation rate of colon cells, being considered an intermediate biomarker of CC risk [48]. Although results did not reach statistical differences, the expression levels of Ntn-1 receptors tended to decrease, suggesting that the downregulation of their ligand effectively modulates the inflammatory response.

Progressive malignancies are characterised by elevated *NTN1* together with decreased expression levels of *DCC* and *UNC5* receptors, which trigger apoptosis via p53 when unbound to NTN-1 [41,49,50,51]. Consequently, either losing receptor activity or increasing *NTN1* expression is advantageous for tumour progression. However, depending on the type of NTN-1 receptor expression, cells may be either attracted or repulsed by NTN-1 with *DCC* and *NEO-1* being classic attractive receptors and the UNC family repulsive receptors [52,53]. In addition, the loss of NTN-1 receptors is not arbitrary and can be attributed to particular stages of tumour growth [54]. Early *UNC5C* inactivation contrasts with late *DCC* loss during multistep CC, while increased *NTN1* mRNA expression has been detected in stages III and IV of CRC [54]. High NTN-1 signalling through *UNC5B* and *DCC* during tumorigenesis has been related to cancer cell proliferation and migration by up-regulation of the proto-oncogene YAP, a downstream signalling of the Hippo pathway, important in cell proliferation and apoptosis [55]. Due to the opposing roles of NTN-1 and its receptors, the development of a humanised monoclonal antibody in phase II clinical trials selectively inhibiting *NTN1* has shed light onto restoring apoptosis in patients with advanced solid tumours, including CC [56]. In an effort to determine the impact of VAT-derived adipokines from patients with OB on NTN-1 receptors, we stimulated both HT-29 and Caco-2 cells with ACM, showing upregulated *DCC* and *NEO1* mRNA levels, respectively. In parallel, we studied the effect of the exogenous administration of NTN-1 on the regulation of its receptors and found upregulated *DCC* and *NEO1* mRNA levels in HT-29 cells and increased *DCC* levels in Caco-2 cells, suggesting that increased NTN-1 in CC and NTN-1-rich VAT media derived from patients with OB might induce CC progression mainly through NEO-1 and *DCC* [14]. In addition to cell survival, NTN-1 and its receptors have been shown to regulate cell morphogenesis, motility, and polarisation events [57].

Invasion of CC cells is associated with advanced tumour stages. Increased expression levels of *NTN1* in gastric cancer samples have been found in patients with stage III and IV CRC, indicating that *NTN1* overexpression may promote tumour metastasis. Our data show a greater migratory capacity of Caco-2 cells under ACM and NTN-1 compared to treatment with the proinflammatory cytokine IL-8, highlighting increased motility of Caco-2 cells under the effects of VAT-derived medium from patients with OB rich in NTN-1 and NTN-1 [58]. According to a study on the migratory effect of NTN-1 in a 3D cell culture model of liver cancer cells, NTN-1 increased N-cadherin-mediated cell junctions (a hallmark of epithelial-to-mesenchymal transition) and promoted cell migration, while *NTN1* silencing decreased N-cadherin and inhibited cell migration, revealing that the ability of NTN-1 to enhance migration may also increase the metastasis of liver cancer. In this line, the importance of NTN-1 on the formation of intracellular fibres for cell contraction and motility in a human colon carcinoma cell line has been described, with greater cell retraction and an increase in actin filaments along the cell margins, further supporting the migratory effect of NTN-1 on tumour cells [50].

The study has some limitations. Further studies in larger cohorts will shed light on our understanding of the role of NTN-1 in obesity-associated CC. Future mechanistic studies in animal models may help to better understand the role of this molecule in AT, and the study of the different signalling pathways triggered by NTN-1 in this tissue is a subject of further investigation. Cell cultures are in vitro systems that are different at a molecular level from the in vivo situation and this difference may be reflected in the secretome. In addition, cell cultures do not represent a real tumour as they are cultured as isolated adenocarcinoma cell lines; therefore, the development of co-cultures comprising both tumour cell lines may constitute an optimal strategy to find an in vitro model to mimic the intestinal epithelium.

## 5. Conclusions

**Figure 6:**
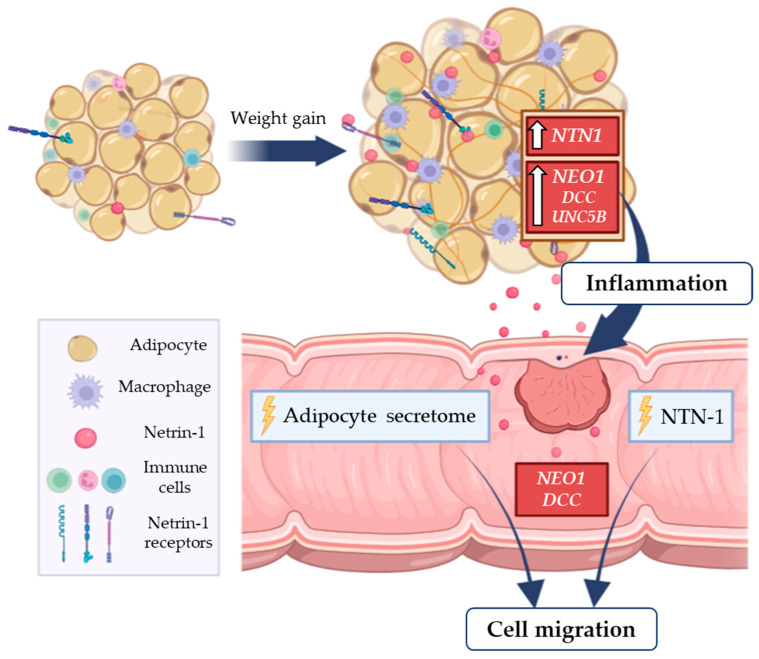
Obesity-associated chronic low-grade inflammation is related with increased risk of colon cancer. In this context, the increased gene expression levels of the pro-inflammatory factor *NTN1* and its receptor *NEO1* in VAT from patients with OB and CC promote a pro-inflammatory microenvironment that may favour tumour progression. The treatment of a colorectal adenocarcinoma cell line with NTN-1 and with the ACM from patients with OB promoted tumour cell migration, emphasising a key role of NTN-1 in CC progression. CC, colon cancer; *DCC*, deleted in colorectal carcinomas; *NEO-1*, neogenin-1; NTN1, netrin-1; OB, obesity; *UNC5B*, uncoordinated-5 homolog B; VAT, visceral adipose tissue.

Collectively, these findings suggest that the upregulated *NTN1* gene expression in VAT from patients with OB and CC promotes a pro-inflammatory microenvironment that may trigger tumour progression and migration (Figure 6). Future studies are required to elucidate the role of NTN-1 in the molecular pathophysiology of CC progression, as well as to assess the impact of its downregulation on obesity-related CC.

## Figures and Tables

**Table 1:** Anthropometric and biochemical characteristics of the volunteers included in the study.

	Non-CC		CC				
	NW	OB	NW	OB	*P* OB	*P* CC	*P* OB × CC
n	9	37	16	12			
Age (years)	44 ± 6.4	46 ± 2.2	60 ± 2.6	64 ± 3.1	0.410	**<0.001**	0.652
Weight (kg)	59.5 ± 2.8	115 ± 3.8 ***	64.5± 2.2 ^†††^	80.3 ± 2.9 **^,†††,#^	**<0.001**	**0.010**	**<0.001**
BMI (kg/m^2^)	20.8 ± 0.5	40.9 ± 1.3 ***	21.8 ± 0.4 ^†††^	29.5 ± 0.7 ***^,†††,###^	**<0.001**	**0.001**	**<0.001**
Estimated BF (%)	22.4 ± 2.1	50.0 ± 1.3 ***	28.3 ± 1.6 ^†††^	35.2 ± 1.7 **^,†††^	**<0.001**	0.076	**<0.001**
Glucose (mg/dL)	88 ± 4.6	119.9 ± 8.4	130.2 ± 14.9	127.5 ± 8.8	0.341	0.217	0.105
Triglycerides (mg/dL)	71 ± 11.1	131.9 ± 9.7	94.2 ± 21.8	182 ± 19	**0.005**	0.382	0.569
Leucocytes (×10^6^)	6.4 ± 0.5	7.3 ± 0.4	6.8 ± 1	7.7 ± 0.8	0.542	0.524	0.507
S100A8 (ng/mL)	357.4 ± 93.6	552.9 ± 69	233 ± 34.8	387.9 ± 57.4	**<0.001**	0.591	0.372
Lactoferrin (ng/mL)	37.9 ± 5.7	71.3 ± 7.7	50.8 ± 7.2	55.8 ± 7.6	**0.037**	0.981	0.197
CCL5 (ng/mL)	5.1 ± 0.7	9.3 ± 0.7 **	6.6 ± 0.7 ^†^	5.4 ± 0.7 ^††^	**0.048**	**0.043**	**0.002**

BMI, body mass index; BF, body fat; CC, colon cancer; CCL5, C-C chemokine ligand 5; NW, normal weight; OB, obesity; S100A8, S100 calcium-binding protein A8. Data are mean ± SEM. Differences between groups were analysed by two-way ANCOVA and one-way ANOVA followed by Tukey’s post hoc tests as appropriate. ** *p* < 0.01 and *** *p* < 0.001 vs. NW non-CC; ^†^ *p* < 0.05, ^††^ *p* < 0.01 and ^†††^ *p* < 0.001 vs. OB non-CC; ^#^ *p* < 0.05 and ^###^ *p* < 0.001 vs. NW CC. *p* values lower than 0.05 are highlighted in bold.

**Table 2:** Analysis of the correlation between gene expression levels of *NTN1*, its receptors and inflammation-related genes in visceral adipose tissue.

	mRNA *NTN1*		mRNA *NEO1*		mRNA *DCC*		mRNA *UNC5B*	
	r	*P*	r	*P*	r	*P*	r	*P*
mRNA *NTN1*	-	-	0.69	**<0.001**	0.32	**0.011**	0.37	**0.004**
mRNA *NEO1*	0.69	**<0.001**	-	-	0.37	**0.003**	0.49	**<0.001**
mRNA *DCC*	0.32	**0.011**	0.37	**0.003**	-	**-**	0.47	**<0.001**
mRNA *UNC5B*	0.37	**0.004**	0.49	**<0.001**	0.47	**0.000**	-	-
mRNA *ASC*	0.40	**<0.001**	0.57	**<0.001**	0.13	0.307	0.41	**<0.001**
mRNA *IL1B*	0.03	0.811	0.21	0.128	0.20	0.146	−0.04	0.756
mRNA *IL18*	0.43	**<0.001**	0.54	**<0.001**	0.16	0.225	0.43	**<0.001**
mRNA *NLRP6*	−0.07	0.59	−0.02	0.872	0.07	0.610	0.06	0.656

Values are Pearson’s correlation coefficients and associated *p* values. *ASC*, apoptosis-associated speck-like protein containing a CARD; *DCC*, deleted in colorectal carcinomas; *IL1B*, interleukin 1 beta; *IL18*, interleukin 18; *NEO1*, neogenin-1; *NLRP6*, NLR family pyrin domain containing 6; *NTN1*, netrin-1; *UNC5B*, uncoordinated-5 homolog B. Bold number indicates statistically significant values.

**Table 3:** Gene expression levels of inflammation-related factors in human visceral adipose tissue.

	Non CC		CC				
	NW	OB	NW	OB	*P* OB	*P* CC	*P* OB × CC
*ASC*	1.00 ± 0.23	1.46 ± 0.10 **	1.62 ± 0.19 **	1.61 ± 0.29 *	**0.003**	**0.001**	**0.010**
*IL1B*	1.00 ± 0.31	5.71 ± 0.26	5.34 ± 2.04	8.49 ± 2.61	**0.016**	**0.022**	0.439
*IL18*	1.00 ± 0.28	0.95 ± 0.12	1.41 ± 0.23	1.14 ± 0.36	0.026	0.004	**0.024**
*NLRP6*	1.00 ± 0.23	6.94 ± 1.36	6.00 ± 1.63	9.61 ± 2.94	**0.006**	**0.004**	0.137

*ASC*, apoptosis-associated speck-like protein containing a CARD; CC, colorectal cancer; *IL*, interleukin; NW, normal weight; *NLRP6*, NLR family pyrin domain containing 6; OB, obesity. Data are mean ± SEM. Differences between groups were analysed by two-way ANCOVA and one-way ANOVA followed by Tukey’s post hoc tests as appropriate. Bold number indicates statistically significant values. * *p* < 0.05 and ** *p* < 0.01 vs. LN non-CC.

## Data Availability

The data that support the findings of this study are available from the corresponding author upon reasonable request.

## Supplementary Materials

The following supporting information can be downloaded at: https://www.mdpi.com/article/10.3390/cancers15041038/s1, Table S1: Clinicopathological characteristics of patients with CC. Table S2: Sequences of the primers and TaqMan^®^ probes.

## References

[1] Dekker E., Tanis P.J., Vleugels J.L.A., Kasi P.M., Wallace M.B. (2019). Colorectal cancer. Lancet.

[2] Siegel R.L., Miller K.D., Goding Sauer A., Fedewa S.A., Butterly L.F., Anderson J.C., Cercek A., Smith R.A., Jemal A. (2020). Colorectal cancer statistics, 2020. CA Cancer J. Clin..

[3] Afshin A., Forouzanfar M.H., Reitsma M.B., Sur P., Estep K., Lee A., Marczak L., Mokdad A.H., Moradi-Lakeh M., GBD 2015 Obesity Collaborators (2017). Health effects of overweight and obesity in 195 countries over 25 years. N. Engl. J. Med..

[4] Lauby-Secretan B., Scoccianti C., Loomis D., Grosse Y., Bianchini F., Straif K. (2016). Body fatness and cancer--viewpoint of the IARC working group. N. Engl. J. Med..

[5] Frühbeck G., Busetto L., Dicker D., Yumuk V., Goossens G.H., Hebebrand J., Halford J.G., Farpour-Lambert N.J., Blaak E.E., Woodward E. (2019). The ABCD of Obesity: An EASO Position Statement on a Diagnostic Term with Clinical and Scientific Implications. Obes. Facts.

[6] Quail D.F., Dannenberg A.J. (2019). The obese adipose tissue microenvironment in cancer development and progression. Nat. Rev. Endocrinol..

[7] Pérez-Hernández A.I., Catalán V., Gómez-Ambrosi J., Rodríguez A., Frühbeck G. (2014). Mechanisms linking excess adiposity and carcinogenesis promotion. Front. Endocrinol..

[8] Xia X., Hu Z., Wang S., Yin K. (2022). Netrin-1: An emerging player in inflammatory diseases. Cytokine Growth Factor Rev..

[9] Ziegon L., Schlegel M. (2021). Netrin-1: A modulator of macrophage driven acute and chronic inflammation. Int. J. Mol. Sci..

[10] Mazelin L., Bernet A., Bonod-Bidaud C., Pays L., Arnaud S., Gespach C., Bredesen D.E., Scoazec J.Y., Mehlen P. (2004). Netrin-1 controls colorectal tumorigenesis by regulating apoptosis. Nature.

[11] Ylivinkka I., Keski-Oja J., Hyytiäinen M. (2016). Netrin-1: A regulator of cancer cell motility?. Eur J Cell Biol.

[12] Arakawa H. (2004). Netrin-1 and its receptors in tumorigenesis. Nat. Rev. Cancer.

[13] Sharma M., Schlegel M., Brown E.J., Sansbury B.E., Weinstock A., Afonso M.S., Corr E.M., van Solingen C., Shanley L.C., Peled D. (2019). Netrin-1 Alters Adipose Tissue Macrophage Fate and Function in Obesity. Immunometabolism.

[14] Mentxaka A., Gómez-Ambrosi J., Ramírez B., Rodríguez A., Becerril S., Neira G., Valentí V., Moncada R., Silva C., Unamuno X. (2022). Netrin-1 promotes visceral adipose tissue inflammation in obesity and is associated with insulin resistance. Nutrients.

[15] Ramkhelawon B., Hennessy E.J., Ménager M., Ray T.D., Sheedy F.J., Hutchison S., Wanschel A., Oldebeken S., Geoffrion M., Spiro W. (2014). Netrin-1 promotes adipose tissue macrophage retention and insulin resistance in obesity. Nat. Med..

[16] Fitamant J., Guenebeaud C., Coissieux M.M., Guix C., Treilleux I., Scoazec J.Y., Bachelot T., Bernet A., Mehlen P. (2008). Netrin-1 expression confers a selective advantage for tumor cell survival in metastatic breast cancer. Proc. Natl. Acad. Sci. USA.

[17] Han P., Liu J., Lei Y., Lin Z., Tian D., Yan W. (2019). Netrin-1 promotes the collective cell migration of liver cancer cells in a 3D cell culture model. J. Physiol. Biochem..

[18] Paradisi A., Maisse C., Coissieux M.M., Gadot N., Lépinasse F., Delloye-Bourgeois C., Delcros J.G., Svrcek M., Neufert C., Fléjou J.F. (2009). Netrin-1 up-regulation in inflammatory bowel diseases is required for colorectal Cancer progression. Proc. Natl. Acad. Sci. USA.

[19] Li B., Shen K., Zhang J., Jiang Y., Yang T., Sun X., Ma X., Zhu J. (2020). Serum netrin-1 as a biomarker for colorectal cancer detection. Cancer Biomark..

[20] Kefeli U., Yildirim M.E., Aydin D., Madenci O.C., Yasar N., Sener N., Mert A.G., Yuksel S., Ercelep O.B., Korkmaz T. (2012). Netrin-1 concentrations in patients with advanced gastric cancer and its relation with treatment. Biomarkers.

[21] Paradisi A., Maisse C., Bernet A., Coissieux M.M., Maccarrone M., Scoazec J.Y., Mehlen P. (2008). NF-KappaB regulates netrin-1 expression and affects the conditional tumor suppressive activity of the netrin-1 receptors. Gastroenterology.

[22] Paradisi A., Mehlen P. (2010). Netrin-1, a missing link between chronic inflammation and tumor progression. Cell Cycle.

[23] Castets M., Broutier L., Molin Y., Brevet M., Chazot G., Gadot N., Paquet A., Mazelin L., Jarrosson-Wuilleme L., Scoazec J.-Y. (2011). DCC constrains tumour progression via its dependence receptor activity. Nature.

[24] Frühbeck G., Catalán V., Rodríguez A., Ramírez B., Becerril S., Salvador J., Colina I., Gómez-Ambrosi J. (2019). Adiponectin-leptin ratio is a functional biomarker of adipose tissue inflammation. Nutrients.

[25] Gómez-Ambrosi J., Silva C., Catalán V., Rodríguez A., Galofré J.C., Escalada J., Valentí V., Rotellar F., Romero S., Ramírez B. (2012). Clinical usefulness of a new equation for estimating body fat. Diabetes Care.

[26] Rodríguez A., Gomez-Ambrosi J., Catalan V., Rotellar F., Valentí V., Silva C., Mugueta M.D.C., Pulido M.R., Vázquez R., Salvador J. (2012). The ghrelin O-acyltransferase–ghrelin system reduces TNF-α-induced apoptosis and autophagy in human visceral adipocytes. Diabetologia.

[27] Muruzábal F.J., Frühbeck G., Gómez-Ambrosi J., Archanco M., Burrell M.A. (2002). Immunocytochemical detection of leptin in non-mammalian vertebrate stomach. Gen. Comp. Endocrinol..

[28] Rodríguez A., Catalán V., Gómez-Ambrosi J., García-Navarro S., Rotellar F., Valentí V., Silva C., Gil M.J., Salvador J., Burrell M.A. (2011). Insulin- and leptin-mediated control of aquaglyceroporins in human adipocytes and hepatocytes is mediated via the PI3K/Akt/MTOR signaling cascade. J. Clin. Endocrinol. Metab..

[29] Frühbeck G., Mentxaka A., Ahechu P., Gómez-Ambrosi J., Ramírez B., Becerril S., Rodríguez A., Unamuno X., Cienfuegos J.A., Casado M. (2021). The differential expression of the inflammasomes in adipose tissue and colon influences the development of colon cancer in a context of obesity by regulating intestinal inflammation. J. Inflamm. Res..

[30] Catalán V., Gómez-Ambrosi J., Rodríguez A., Ramírez B., Ortega V.A., Hernández-Lizoain J.L., Baixauli J., Becerril S., Rotellar F., Valentí V. (2017). IL-32α-induced inflammation constitutes a link between obesity and colon cancer. Oncoimmunology.

[31] Catalán V., Gómez-Ambrosi J., Rotellar F., Silva C., Rodríguez A., Salvador J., Gil M.J., Cienfuegos J.A., Frühbeck G. (2007). Validation of endogenous control genes in human adipose tissue: Relevance to obesity and obesity-associated type 2 diabetes mellitus. Horm. Metab. Res..

[32] Ning Y., Manegold P.C., Hong Y.K., Zhang W., Pohl A., Lurje G., Winder T., Yang D., LaBonte M.J., Wilson P.M. (2011). Interleukin-8 is associated with proliferation, migration, angiogenesis and chemosensitivity in vitro and in vivo in colon cancer cell line models. Int. J. Cancer.

[33] Kuai W.X., Wang Q., Yang X.Z., Zhao Y., Yu R., Tang X.J. (2012). Interleukin-8 associates with adhesion, migration, invasion and chemosensitivity of human gastric cancer cells. World J. Gastroenterol..

[34] Bird C., Kirstein S. (2009). Real-time, label-free monitoring of cellular invasion and migration with the XCELLigence system. Nat. Methods.

[35] Ly N.P., Komatsuzaki K., Fraser I.P., Tseng A.A., Prodhan P., Moore K.J., Kinane T.B. (2005). Netrin-1 inhibits leukocyte migration in vitro and in vivo. Proc. Natl. Acad. Sci. USA.

[36] Martín M., Rodríguez A., Gómez-Ambrosi J., Ramírez B., Becerril S., Catalán V., López M., Diéguez C., Frühbeck G., Burrel M.A. (2021). Caloric restriction prevents metabolic dysfunction and the changes in hypothalamic neuropeptides associated with obesity independently of dietary fat content in rats. Nutrients.

[37] Nieman K.M., Romero I.L., Van Houten B., Lengyel E. (2013). Adipose tissue and adipocytes support tumorigenesis and metastasis. Biochim. Biophys. Acta.

[38] Rajesh Y., Sarkar D. (2021). Association of adipose tissue and adipokines with development of obesity-induced liver cancer. Int. J. Mol. Sci..

[39] Zhang X., Cui P., Ding B., Guo Y., Han K., Li J., Chen H., Zhang W. (2018). Netrin-1 elicits metastatic potential of non-small cell lung carcinoma cell by enhancing cell invasion, migration and vasculogenic mimicry via EMT induction. Cancer Gene Ther..

[40] Chen H., Chen Q., Luo Q. (2016). Expression of netrin-1 by hypoxia contributes to the invasion and migration of prostate carcinoma cells by regulating YAP activity. Exp. Cell Res..

[41] Kefeli U., Ucuncu Kefeli A., Cabuk D., Isik U., Sonkaya A., Acikgoz O., Ozden E., Uygun K. (2017). Netrin-1 in cancer: Potential biomarker and therapeutic target?. Tumor Biol..

[42] Frühbeck G. (2005). Obesity: Aquaporin enters the picture. Nature.

[43] Catalan V., Gómez-Ambrosi J., Rodríguez A., Ramírez B., Rotellar F., Valentí V., Silva C., Gil M.J., Fernández-Real J.M., Salvador J. (2011). Increased Levels of Calprotectin in Obesity Are Related to Macrophage Content: Impact on Inflammation and Effect of Weight Loss. Mol. Med..

[44] Fantuzzi G. (2005). Adipose tissue, adipokines, and inflammation. J. Allergy Clin. Immunol..

[45] Ringel A.E., Drijvers J.M., Baker G.J., Catozzi A., García-Cañaveras J.C., Gassaway B.M., Miller B.C., Juneja V.R., Nguyen T.H., Joshi S. (2020). Obesity shapes metabolism in the tumor microenvironment to suppress anti-tumor immunity. Cell.

[46] Meynet O., Ricci J.E. (2014). Caloric restriction and cancer: Molecular mechanisms and clinical implications. Trends Mol. Med..

[47] Olivo-Marston S.E., Hursting S.D., Perkins S.N., Schetter A., Khan M., Croce C., Harris C.C., Lavigne J. (2014). Effects of calorie restriction and diet-induced obesity on murine colon carcinogenesis, growth and inflammatory factors, and microRNA expression. PLoS ONE.

[48] Steinbach G., Kumar S.P., Reddy B.S., Lipkin M., Holt P.R. (1993). Effects of caloric restriction and dietary fat on epithelial cell proliferation in rat colon. Cancer Res..

[49] Grandin M., Meier M., Delcros J.G., Nikodemus D., Reuten R., Patel T.R., Goldschneider D., Orriss G., Krahn N., Boussouar A. (2016). Structural decoding of the netrin-1/UNC5 interaction and its therapeutical implications in cancers. Cancer Cell.

[50] Rodrigues S., De Wever O., Bruyneel E., Rooney R.J., Gespach C. (2007). Opposing roles of netrin-1 and the dependence receptor DCC in cancer cell invasion, tumor growth and metastasis. Oncogene.

[51] Mehlen P., Llambi F. (2005). Role of netrin-1 and netrin-1 dependence receptors in colorectal cancers. Br. J. Cancer.

[52] Boyer N.P., Gupton S.L. (2018). Revisiting netrin-1: One who guides (axons). Front. Cell. Neurosci..

[53] Untiveros G., Raskind A., Linares L., Dotti A., Strizzi L. (2022). Netrin-1 stimulates migration of neogenin expressing aggressive melanoma cells. Int. J. Mol. Sci..

[54] Shin S.K., Nagasaka T., Jung B.H., Matsubara N., Kim W.H., Carethers J.M., Boland C.R., Goel A. (2007). Epigenetic and genetic alterations in netrin-1 receptors UNC5C and DCC in human colon cancer. Gastroenterology.

[55] Qi Q., Li D.Y., Luo H.R., Guan K.L., Ye K. (2015). Netrin-1 exerts oncogenic activities through enhancing yes-associated protein stability. Proc. Natl. Acad. Sci. USA.

[56] First in Human Evaluation of Safety, Pharmacokinetics, and Clinical Activity of a Monoclonal Antibody Targeting Netrin-1 in Patients with Advanced/Metastatic Solid Tumors (NP137). https://clinicaltrials.gov/ct2/show/NCT02977195.

[57] Murray M.J. (2017). The role of netrins and their receptors in epithelial mesenchymal plasticity during development. Cells Tissues Organs.

[58] Sturm A., Baumgart D.C., d’Heureuse J.H., Hotz A., Wiedenmann B., Dignass A.U. (2005). CXCL8 modulates human intestinal epithelial cells through a CXCR1 dependent pathway. Cytokine.

