# Supplementary material for: Increased Expression Levels of Netrin-1 in Visceral Adipose Tissue during Obesity Favour Colon Cancer Cell Migration
Source: Cancers (Basel). 2023 Feb 7;15(4):1038. doi: 10.3390/cancers15041038 (PMC9953821; doi:10.3390/cancers15041038)
Supplement: Supplementary file 1 [file cancers-15-01038-s001.zip › cancers-2124596-supplementary.pdf]

Table S1. Clinicopathological characteristics of patients with colon cancer.

**Gender, n**	
Male	17
Female	11
**Location of primary lesion, n**	
Transverse colon	4
Right hemicolon	11
Left hemicolon	11
Missing	2
**TNM stage, n**	
I	4
II	5
III	13
IV	4
Missing	2
**Differentiation, n**	
Well	3
Moderately	20
Poorly and undifferentiated	3
Missing	2
**Tumor size, n**	
< 5 cm	15
> 5 cm	7
Missing	6
**Lymph node status, n**	
Positive	8
Negative	20

Table S2. Sequences of primers and TaqMan® probes.

Gene (GenBank accession)	Oligonucleotide sequence (5′-3′)
ASC (NM_013258.4)	
Forward	AGCCAGGCCTGCACTTTATAGA
Reverse	CAGCAGCCACTCAACGTTTG
TaqMan® Probe	FAM-CACCGGGCTGCGCTTATCGC-TAMRA
DCC (NM_005215.4)	
Forward	ACCCTAAATGAGCCGCCAAT
Reverse	GACACCAACGGTGACCACAAT
TaqMan® Probe	FAM-CACTCCTCAGAAGAACAGCAACCTGCTTGT -TAMRA
IL1B (NM_000576)	
Forward	CAGTGGCAATGAGGATGACTTG
Reverse	GTAGTGGTGGTCGGAGATTCGTA
TaqMan® Probe	FAM-TGGCCCTAAACAGATGAAGTGCTCCTTCC-TAMRA
IL18 (NM_001562)	
Forward	CCAAGGAAATCGGCCTCTATT
Reverse	CCTCTAGGCTGGCTATCTTTATACATACT
TaqMan® Probe	FAM-TTCTGACTGTAGAGATAATGCACCCCGGAC-TAMRA
NEO1 (NM_001172623.1)	
Forward	GTGGAGCCCAACTGATAATCCTT
Reverse	CAATTTGATGAAGCGGGTAGAGA
TaqMan® Probe	FAM-AACATGCACCAGCCACAACGGGA-TAMRA
NLRP6 (NM_138329.2)	
Forward	TCCCTTCTTCATCCACTCTTTCAG
Reverse	CAGACCGCGTCAGGGAGTT
TaqMan® Probe	FAM-CTGAGCAGCCTCACGCTGTCCCA-TAMRA
NTN1 (NM_000575)	
Forward	AGGGCTACTACCGCGACATG
Reverse	ACACTGGCCGGTGGTTTG
TaqMan® Probe	FAM-TGCAAAGCCTGTGATTGCCACCC-TAMRA
UNC5B (NM_001244889.2)	
Forward	AGAGTCGCCGAGCCTACGT
Reverse	TTGCCCAGAGGCTCCTGAT
TaqMan® Probe	FAM-CGCATCGCCTACCTGCGCAAG-TAMRA
r*Neo1* (XM_039082421.1)	
Forward	AGGCCCTTGTGACAGTTCCA
Reverse	AATCCCTTTGTCCTGTTCAAGAGA
TaqMan® Probe	FAM-AGCCACAGAGTAACCCCAGTCAGCGTG-TAMRA
r*Ntn1* (NM_053731.2)	
Forward	AGGCCCTTGTGACAGTTCCA
Reverse	AATCCCTTTGTCCTGTTCAAGAGA
TaqMan® Probe	FAM-AGCCACAGAGTAACCCCAGTCAGCGTG-TAMRA

ASC, apoptosis-associated speck-like protein containing a CARD; *DCC*, deleted in colorectal cancer; *IL*, interleukin; *NEO1*, neogenin-1; *NLRP6*, NLR family pyrin domain containing 6; *NTN1*, netrin-1; *UNC5B*, UNC-5 netrin receptor B.
